# 2,3,4,6-Tetra-*O*-acetyl-1-[(dimethylcarbamothioyl)sulfanyl]-*β*-d-galacto­pyran­ose

**DOI:** 10.1107/S2414314625005449

**Published:** 2025-06-24

**Authors:** Reham A. Mohamed-Ezzat, Galal H. Elgemeie, Peter G. Jones

**Affiliations:** a Chemistry of Natural and Microbial Products Department, Pharmaceutical and Drug Industries Research Institute, National Research Centre, Cairo, Egypt; b Chemistry Department, Faculty of Science, Helwan University, Cairo, Egypt; c Institut für Anorganische und Analytische Chemie, Technische Universität Braunschweig, Hagenring 30, D-38106 Braunschweig, Germany; Goethe-Universität Frankfurt, Germany

**Keywords:** thio­carbamate, galactose, weak hydrogen bonds, crystal structure

## Abstract

In the structure of the title compound, C_17_H_25_NO_9_S_2_, the bond lengths in the C—S—C moiety are almost equal at 1.7959 (8) and 1.7877 (9) Å, with a shorter formally double C—S bond of 1.6698 (9) Å at the other sulfur atom. The eight-atom sequence O3—C3—C2—C1—S—C—N—C (using standard sugar numbering) shows an extended conformation. The packing involves ‘weak’ hydrogen bonds, whereby the three shortest C—H⋯O contacts combine to form layers of mol­ecules parallel to the *ab* plane.

## Abstract 2

The bond lengths in the C—S—C moiety are almost equal, with a shorter formally double C—S bond. The packing involves ‘weak’ hydrogen bonds; the three shortest contacts combine to form layers parallel to the *ab* plane.

## Graphical abstract

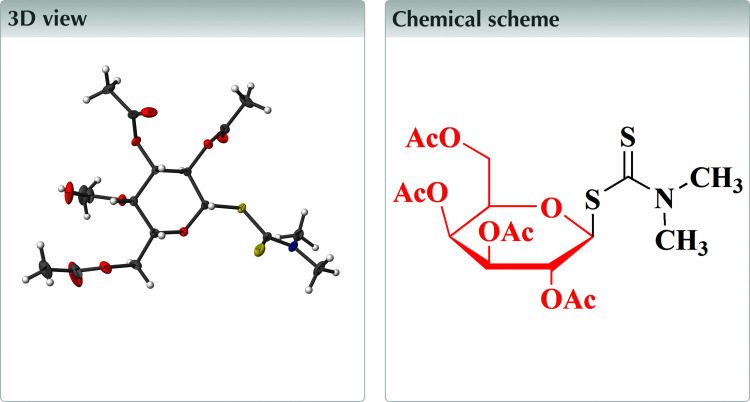

## Structure description

Thio­glycosides have been the focus of much attention because of their role as glycosyl donors in a variety of chemical processes. They can be subjected to most common manipulations of carbohydrate-protecting groups (Toshima *et al.*, 2007 ▸), and can be activated for glycosidation under a variety of conditions. An associated advantage is their stability in such processes (Lian *et al.*, 2015 ▸). Oligosaccharides and glycoconjugates have a wide range of biological roles because of the extensive variety of their mol­ecular structures. They are particularly desirable synthetic targets in terms both of their biological significance and of the synthetic challenges they offer, and synthetic carbohydrate chemistry has long been a major area of inter­est in organic chemistry (Codée *et al.*, 2005 ▸). Additionally, some thio­glycoside derivatives have been reported to be inhibitors of protein glycosyl­ation (Scala *et al.*, 1997 ▸).

We have reported the structures of several thio­glycosides, the most recent being four structures involving carbamimido­thio­ate groups (Abu-Zaied *et al.*, 2024 ▸; see also references therein). Here, we report the structure of *N,N*-di­methyl­carbamodi­thio­(2,3,4,6-tetra-*O*-acetyl-*β*-d-galacto­pyran­ose), made by reacting potassium cyano­carbonimidodi­thio­ate with the protected α-d-galacto­pyranosyl bromide in dimethyl formamide in the presence of sodium ethoxide at room temperature for 24 h. The compound has been previously reported by Li *et al.* (2016 ▸), Pluigers *et al.* (1969 ▸), Ferrier & Furneaux (1977 ▸) and Tejima & Ishiguro (1967 ▸).

**Figure 1:**
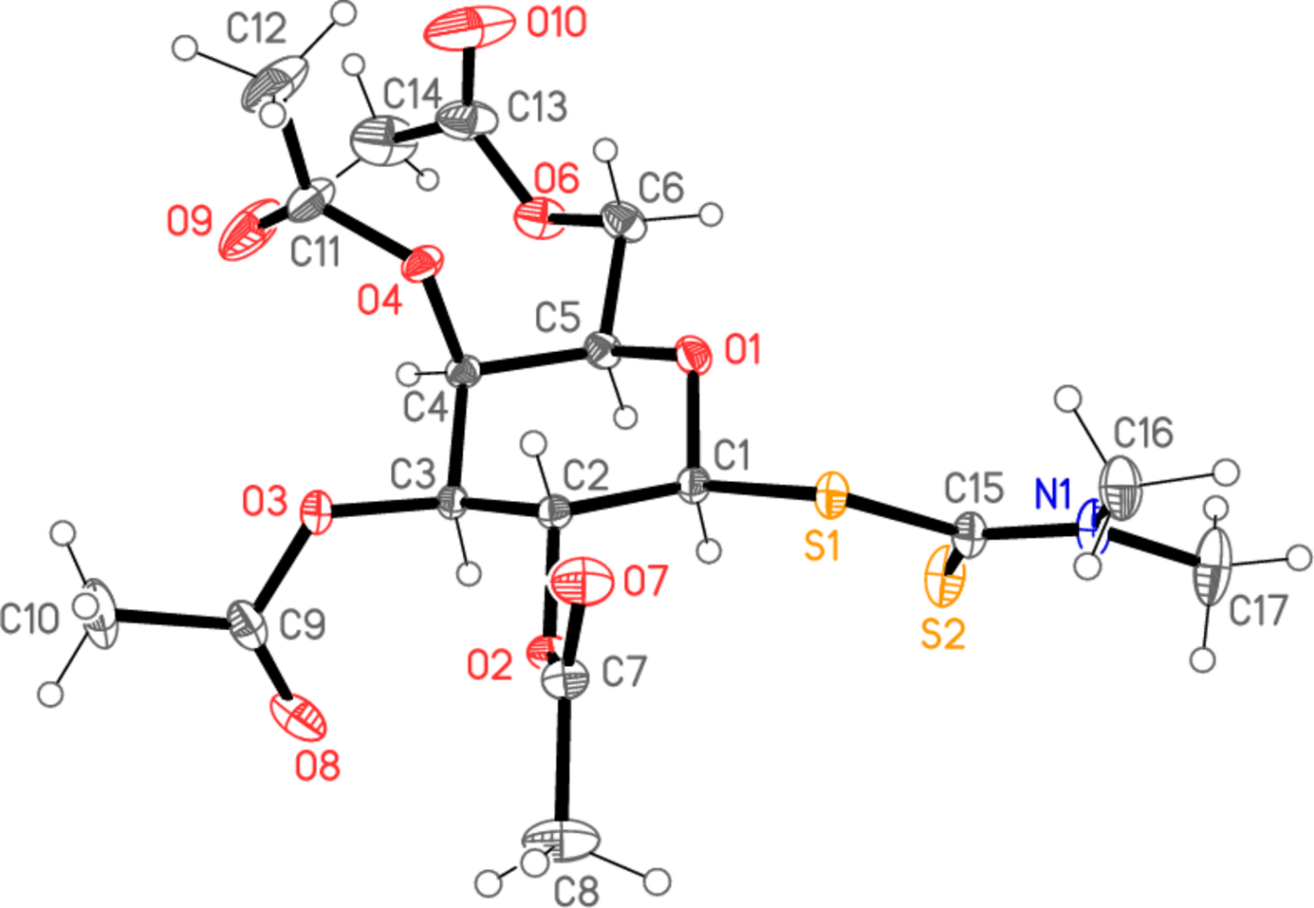
The mol­ecule of the title compound in the crystal. Ellipsoids indicate 50% probability levels.

The mol­ecule of the title compound is shown in Fig. 1 ▸, with selected mol­ecular dimensions in Table 1 ▸. Bond lengths and angles may be considered normal, *e.g.* the two almost equal C—S1 bond lengths and the shorter S2—C15, corresponding to its formal double bond nature. The atom sequence O3—C3—C2—C1—S1—C15—N1—C17 shows an extended conformation, with absolute torsion angles 155.53 (6)° for C2—C1—S1—C15 (confirming the β position of the substituent at C1) and > 170° for all others. The geometry at the nitro­gen atom is planar (angle sum 359.9°).

**Figure 2:**
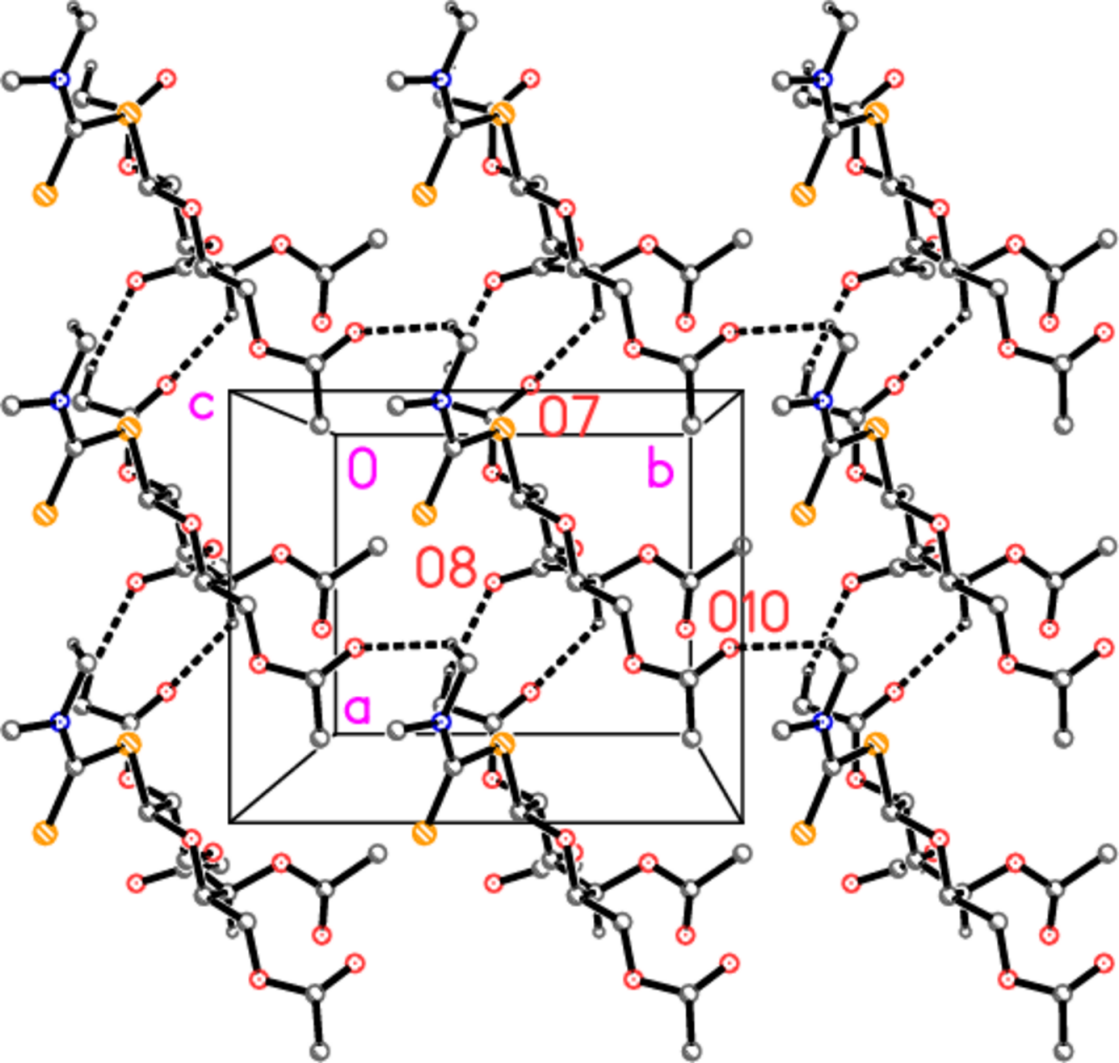
Packing diagram of the title compound, viewed parallel to the *c* axis, showing the layer at *z* ≃ 0.25. Dashed lines indicate C—H⋯O hydrogen bonds. Hydrogen atoms not involved in the hydrogen bonds are omitted for clarity. Atoms labels correspond to the asymmetric unit.

**Figure 3:**
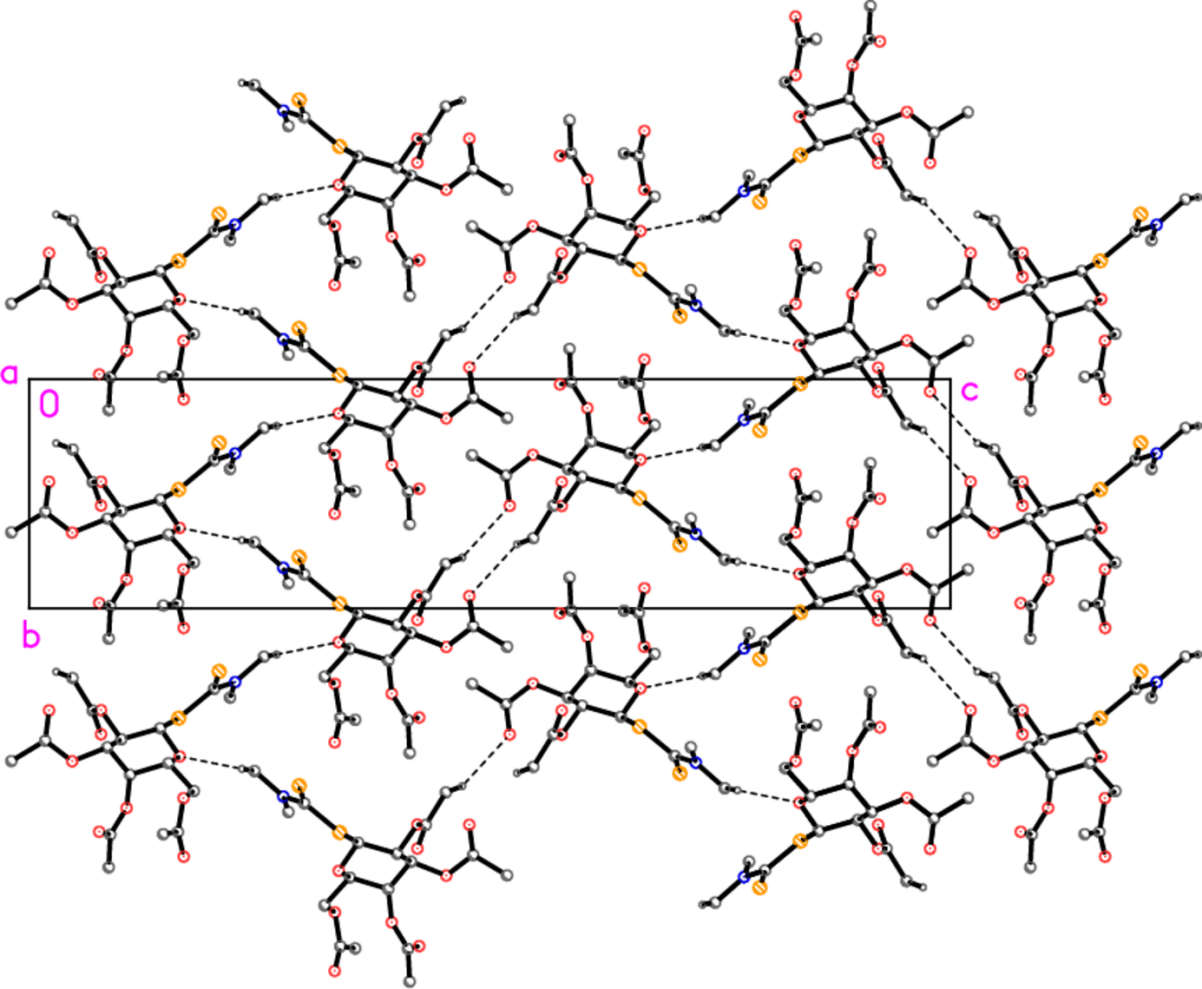
Packing diagram of the title compound, projected parallel to the *a* axis, showing the links between the layers of Fig. 2 ▸. Dashed lines indicate C—H⋯O hydrogen bonds. Hydrogen atoms not involved in the hydrogen bonds are omitted for clarity.

In the absence of classical hydrogen bond donors, the packing involves ‘weak’ hydrogen bonds. The three shortest C—H⋯O contacts (Table 2 ▸) combine to form layers of mol­ecules parallel to the *ab* plane at *z* = 1/4, 1/2, 3/4, *etc*. (Fig. 2 ▸). Layers are linked by the other two C—H⋯O contacts (Fig. 3 ▸).

**Figure 4:**
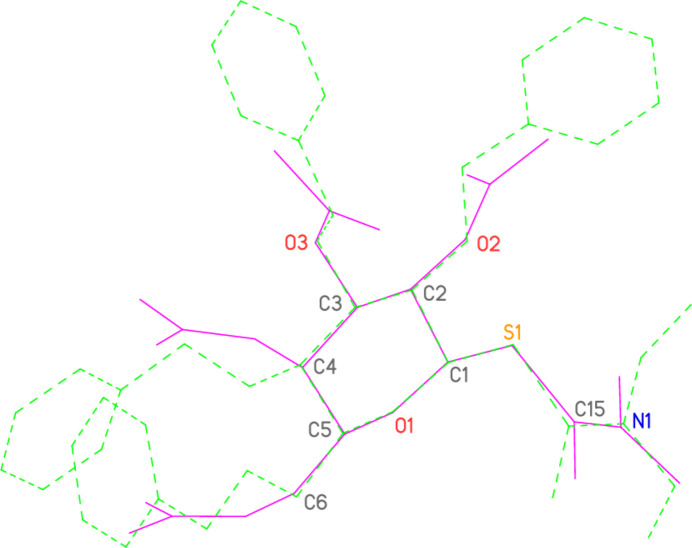
Least-squares fit of the title compound (purple) to YIYKEY (Padungros *et al.*, 2014 ▸) (green; coordinates taken from the CCDC). Fitted atoms are labelled.

A search employing the routine CONQUEST (Bruno *et al.*, 2002 ▸), part of Version 2024.3.0 of the Cambridge Database (Groom *et al.*, 2016 ▸), found only one other pyran­ose sugar with a di­thio­carbamate substituent at the 1-position, namely 1-(*N,N*-di­ethyl­dithio­carbamato)-2,3,4,6-tetra-*O*-benzyl-*β*-d-gluco­pyran­ose (refcode YIYKEY; Padungros *et al.*, 2014 ▸). A least-squares fit of 13 selected atoms in or near the sugar rings of both mol­ecules (Fig. 4 ▸) was performed. In view of the markedly different protecting groups of the sugar rings, together with the opposite configurations of glucose and galactose at C4, no great similarity should be expected, but the the r.m.s. deviation of the fitted atoms is still quite low at 0.08 Å. The deviation for S2, the terminal sulfur atom of the di­thio­carbamate, is appreciably higher at 0.69 Å, reflecting the slightly larger torsion angles C2—C1—S1—C15 and C1—S1—C15—S2 (162.1 and 3.0°, respectively) for YIYKEY.

## Synthesis and crystallization

A mixture of potassium cyano­carbonimidodi­thio­ate (0.01 mol, 1.94 g m) and 2,3,4,6-tetra-*O*-acetyl-*β*-d-galacto­pyranosyl bromide (0.01 mol, 4.11 g m) was reacted in dimethyl formamide (10 ml) in the presence of sodium ethoxide (0.01 mole, 0.68 g m) at room temperature for 24 h. Ice–water (10 ml) was then added and the solid product thus furnished was filtered off and recrystallized from dimethyl sulfoxide.

The title compound was obtained as a pale-yellow crystalline solid; m.p. 458–459 K; ^1^H NMR (500 MHz, DMSO-*d*_6_): δ 1.90, 1.94, 1.98, 2.09 (4 s, 12H, 4OAc), 3.29 (*s*, 3H, CH_3_), 3.42 (*s*, 3H, CH_3_), 3.93–3.96 (*m*, 2H, H-6), 4.25 (*t*, 1H, H-5), 5.22 (*t*, 1H, H-4), 5.28 (*t*, 1H, H-3), 5.36 (*t*, 1H, H-2), 5.79 (*d*, *J* = 10 Hz, 1H, H-1). Analysis calculated for C_17_H_25_NO_9_S_2_ (451.51): C 45.22, H 5.58, N 3.10; S 14.20. Found: C 45.20, H 5.56, N 3.10, S 14.18%. One large prism was cut to an irregular block for intensity measurements.

## Refinement

Crystal data, data collection and structure refinement details are summarized in Table 3 ▸. Methyl groups were refined as idealized rigid groups allowed to rotate but not tip (AFIX 137), with C—H 0.98, H—C—H 109.5°. Other hydrogen atoms were included using a riding model starting from calculated positions (C—H_methine_ 1.00, *C*—H_methyl­ene_ 0.99 Å). The *U*(H) values were fixed for methyl groups at 1.5 × *U*_eq_, and for other H atoms at 1.2 × *U*_eq_ of the parent carbon atoms. Three badly-fitting reflections (deviations > 8σ) were omitted from the refinement. The absolute configuration was confirmed by the Flack *x* value of 0.001 (8).

## Supplementary Material

Crystal structure: contains datablock(s) I, global. DOI: 10.1107/S2414314625005449/bt4175sup1.cif

Structure factors: contains datablock(s) I. DOI: 10.1107/S2414314625005449/bt4175Isup2.hkl

CCDC reference: 2465327

Additional supporting information:  crystallographic information; 3D view; checkCIF report

## Figures and Tables

**Table 1:** Selected geometric parameters (Å, °)

C1—S1	1.7959 (8)	S2—C15	1.6698 (9)
S1—C15	1.7877 (9)	C15—N1	1.3327 (12)
			
C15—S1—C1	101.77 (4)	C15—N1—C17	119.92 (9)
N1—C15—S2	124.18 (7)	C15—N1—C16	123.38 (8)
N1—C15—S1	112.09 (7)	C17—N1—C16	116.62 (8)
S2—C15—S1	123.71 (5)		
			
S1—C1—C2—C3	−179.78 (5)	C1—S1—C15—N1	170.41 (7)
C1—C2—C3—O3	−173.98 (6)	C1—S1—C15—S2	−11.04 (7)
C2—C1—S1—C15	155.53 (6)	S1—C15—N1—C17	175.46 (10)

**Table 2:** Hydrogen-bond geometry (Å, °)

*D*—H⋯*A*	*D*—H	H⋯*A*	*D*⋯*A*	*D*—H⋯*A*
C4—H4⋯O7^i^	1.00	2.45	3.2096 (11)	132
C8—H8*B*⋯O8^ii^	0.98	2.41	3.3157 (15)	154
C16—H16*C*⋯O10^iii^	0.98	2.44	3.1795 (18)	132
C17—H17*B*⋯O1^iv^	0.98	2.55	3.3558 (13)	139
C1—H1⋯S2	1.00	2.56	3.1175 (8)	115
C8—H8*A*⋯O8^v^	0.98	2.66	3.3384 (15)	127

Symmetry codes: (i) 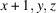; (ii) 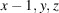; (iii) 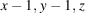; (iv) 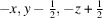; (v) 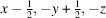.

**Table 3:** Experimental details

Crystal data	
Chemical formula	C_17_H_25_NO_9_S_2_
*M*_r_	451.50
Crystal system, space group	Orthorhombic, *P*2_1_2_1_2_1_
Temperature (K)	100
*a*, *b*, *c* (Å)	7.28265 (10), 8.64720 (15), 34.8789 (3)
*V* (Å^3^)	2196.48 (5)
*Z*	4
Radiation type	Mo *K*α
μ (mm^−1^)	0.29
Crystal size (mm)	0.22 × 0.20 × 0.15

Data collection	
Diffractometer	XtaLAB Synergy
Absorption correction	Multi-scan (*CrysAlis PRO*; Rigaku OD, 2022 ▸)
*T*_min_, *T*_max_	0.818, 1.000
No. of measured, independent and observed [*I* > 2σ(*I*)] reflections	225004, 14433, 13593
*R*_int_	0.045
θ values (°)	θ_max_ = 41.4, θ_min_ = 2.3
(sin θ/λ)_max_ (Å^−1^)	0.930

Refinement	
*R*[*F*^2^ > 2σ(*F*^2^)], *wR*(*F*^2^), *S*	0.031, 0.083, 1.12
No. of reflections	14433
No. of parameters	268
H-atom treatment	H-atom parameters constrained
Δρ_max_, Δρ_min_ (e Å^−3^)	0.56, −0.27
Absolute structure	Flack *x* determined using 5769 quotients [(*I*^+^)−(*I*^−^)]/[(*I*^+^)+(*I*^−^)] (Parsons *et al.*, 2013 ▸)
Absolute structure parameter	0.001 (8)

Computer programs: *CrysAlis PRO* (Rigaku OD, 2022 ▸), *SHELXT* (Sheldrick, 2015*a* ▸), *SHELXL2019/3* (Sheldrick, 2015*b* ▸), *XP* (Bruker, 1998 ▸) and *publCIF* (Westrip, 2010 ▸).

## full crystallographic data

### 2,3,4,6-Tetra-O-acetyl-1-[(dimethylcarbamothioyl)sulfanyl]-β-D-galactopyranose Crystal data

**Table:**

C_17_H_25_NO_9_S_2_	*D*_x_ = 1.365 Mg m^−^^3^
*M**_r_* = 451.50	Mo *K*α radiation, λ = 0.71073 Å
Orthorhombic, *P*2_1_2_1_2_1_	Cell parameters from 126110 reflections
*a* = 7.28265 (10) Å	θ = 2.3–41.3°
*b* = 8.64720 (15) Å	µ = 0.29 mm^−^^1^
*c* = 34.8789 (3) Å	*T* = 100 K
*V* = 2196.48 (5) Å^3^	Block, colourless
*Z* = 4	0.22 × 0.20 × 0.15 mm
*F*(000) = 952	

### 2,3,4,6-Tetra-O-acetyl-1-[(dimethylcarbamothioyl)sulfanyl]-β-D-galactopyranose Data collection

**Table:**

XtaLAB Synergy diffractometer	14433 independent reflections
Radiation source: micro-focus sealed X-ray tube, PhotonJet (Mo) X-ray Source	13593 reflections with *I* > 2σ(*I*)
Mirror monochromator	*R*_int_ = 0.045
Detector resolution: 10.0000 pixels mm^-1^	θ_max_ = 41.4°, θ_min_ = 2.3°
ω scans	*h* = −13→13
Absorption correction: multi-scan (CrysAlisPro; Rigaku OD, 2022)	*k* = −15→15
*T*_min_ = 0.818, *T*_max_ = 1.000	*l* = −63→63
225004 measured reflections	

### 2,3,4,6-Tetra-O-acetyl-1-[(dimethylcarbamothioyl)sulfanyl]-β-D-galactopyranose Refinement

**Table:**

Refinement on *F*^2^	Hydrogen site location: inferred from neighbouring sites
Least-squares matrix: full	H-atom parameters constrained
*R*[*F*^2^ > 2σ(*F*^2^)] = 0.031	*w* = 1/[σ^2^(*F*_o_^2^) + (0.0464*P*)^2^ + 0.1937*P*] where *P* = (*F*_o_^2^ + 2*F*_c_^2^)/3
*wR*(*F*^2^) = 0.083	(Δ/σ)_max_ = 0.010
*S* = 1.12	Δρ_max_ = 0.56 e Å^−^^3^
14433 reflections	Δρ_min_ = −0.27 e Å^−^^3^
268 parameters	Absolute structure: Flack *x* determined using 5769 quotients [(*I*^+^)-(*I*^-^)]/[(*I*^+^)+(*I*^-^)] (Parsons *et al.*, 2013)
0 restraints	Absolute structure parameter: 0.001 (8)
Primary atom site location: dual	

### 2,3,4,6-Tetra-O-acetyl-1-[(dimethylcarbamothioyl)sulfanyl]-β-D-galactopyranose Fractional atomic coordinates and isotropic or equivalent isotropic displacement parameters (Å^2^)

**Table:**

	*x*	*y*	*z*	*U*_iso_*/*U*_eq_	
C1	0.22016 (11)	0.52540 (10)	0.14258 (2)	0.01348 (11)	
H1	0.301124	0.432274	0.144357	0.016*	
C2	0.20058 (11)	0.57372 (9)	0.10035 (2)	0.01244 (11)	
H2	0.119080	0.666256	0.097869	0.015*	
C3	0.39260 (11)	0.60872 (9)	0.08518 (2)	0.01229 (11)	
H3	0.466514	0.511270	0.084781	0.015*	
C4	0.48993 (11)	0.72864 (9)	0.10985 (2)	0.01316 (10)	
H4	0.620302	0.739994	0.101305	0.016*	
C5	0.48404 (12)	0.67805 (10)	0.15167 (2)	0.01507 (12)	
H5	0.557771	0.581254	0.154690	0.018*	
C6	0.55721 (13)	0.80002 (13)	0.17912 (3)	0.02065 (15)	
H6A	0.482407	0.895388	0.177473	0.025*	
H6B	0.553206	0.761446	0.205830	0.025*	
C7	−0.05084 (12)	0.44818 (10)	0.06923 (3)	0.01588 (12)	
C8	−0.10298 (17)	0.30603 (14)	0.04748 (4)	0.0290 (2)	
H8A	−0.004042	0.278698	0.029677	0.044*	
H8B	−0.216034	0.325520	0.033023	0.044*	
H8C	−0.123073	0.220596	0.065454	0.044*	
C9	0.44387 (13)	0.57971 (13)	0.01780 (3)	0.02005 (15)	
C10	0.4443 (2)	0.6688 (2)	−0.01898 (3)	0.0349 (3)	
H10A	0.318449	0.699657	−0.025372	0.052*	
H10B	0.493721	0.603806	−0.039557	0.052*	
H10C	0.520872	0.761240	−0.016113	0.052*	
C11	0.48662 (15)	0.99031 (11)	0.08844 (4)	0.02412 (18)	
C12	0.3713 (2)	1.13341 (14)	0.08674 (6)	0.0395 (4)	
H12A	0.250639	1.108055	0.075993	0.059*	
H12B	0.431714	1.210509	0.070465	0.059*	
H12C	0.355954	1.175358	0.112643	0.059*	
C13	0.79252 (16)	0.97920 (15)	0.16185 (5)	0.0314 (2)	
C14	0.98327 (19)	0.9884 (2)	0.14586 (6)	0.0408 (3)	
H14A	1.061908	0.913046	0.158930	0.061*	
H14B	1.032239	1.092733	0.149860	0.061*	
H14C	0.980359	0.965477	0.118356	0.061*	
O1	0.29972 (9)	0.64895 (8)	0.16373 (2)	0.01595 (10)	
O2	0.13054 (9)	0.44673 (7)	0.07833 (2)	0.01412 (9)	
O3	0.38060 (11)	0.67005 (8)	0.04701 (2)	0.01719 (11)	
O4	0.39544 (9)	0.87439 (7)	0.10612 (2)	0.01611 (10)	
O6	0.74333 (11)	0.83094 (10)	0.16784 (3)	0.02342 (14)	
O7	−0.15325 (10)	0.55175 (9)	0.07833 (3)	0.02197 (13)	
O8	0.49188 (14)	0.44747 (11)	0.02195 (2)	0.02792 (16)	
O9	0.64113 (15)	0.97836 (12)	0.07640 (4)	0.0420 (3)	
O10	0.69249 (18)	1.08682 (14)	0.16806 (6)	0.0599 (5)	
S1	−0.00073 (3)	0.48124 (3)	0.16268 (2)	0.01509 (4)	
S2	0.26816 (3)	0.27711 (4)	0.20661 (2)	0.02352 (5)	
C15	0.05929 (12)	0.35345 (11)	0.20097 (2)	0.01559 (12)	
N1	−0.08300 (11)	0.32376 (11)	0.22384 (2)	0.01961 (13)	
C16	−0.26558 (14)	0.39147 (15)	0.21834 (3)	0.02370 (18)	
H16A	−0.254152	0.503647	0.215141	0.036*	
H16B	−0.342140	0.369253	0.240783	0.036*	
H16C	−0.322539	0.346774	0.195424	0.036*	
C17	−0.06368 (18)	0.2123 (2)	0.25515 (4)	0.0335 (3)	
H17A	−0.028969	0.111306	0.244663	0.050*	
H17B	−0.180709	0.203067	0.268826	0.050*	
H17C	0.031589	0.247892	0.272924	0.050*	

### 2,3,4,6-Tetra-O-acetyl-1-[(dimethylcarbamothioyl)sulfanyl]-β-D-galactopyranose Atomic displacement parameters (Å^2^)

**Table:**

	*U*^11^	*U*^22^	*U*^33^	*U*^12^	*U*^13^	*U*^23^
C1	0.0139 (3)	0.0139 (3)	0.0126 (2)	−0.0006 (2)	0.0018 (2)	0.0012 (2)
C2	0.0134 (3)	0.0111 (3)	0.0128 (3)	0.0005 (2)	0.0010 (2)	0.0000 (2)
C3	0.0142 (3)	0.0114 (3)	0.0112 (2)	0.0007 (2)	0.0021 (2)	0.00144 (19)
C4	0.0124 (2)	0.0112 (2)	0.0159 (3)	0.0009 (2)	0.0020 (2)	−0.0005 (2)
C5	0.0137 (3)	0.0170 (3)	0.0146 (3)	0.0001 (2)	0.0002 (2)	−0.0010 (2)
C6	0.0169 (3)	0.0255 (4)	0.0195 (3)	−0.0028 (3)	−0.0002 (3)	−0.0068 (3)
C7	0.0145 (3)	0.0150 (3)	0.0181 (3)	0.0008 (2)	−0.0015 (2)	−0.0009 (2)
C8	0.0228 (4)	0.0222 (4)	0.0421 (6)	0.0004 (3)	−0.0091 (4)	−0.0124 (4)
C9	0.0173 (3)	0.0306 (4)	0.0123 (3)	−0.0039 (3)	0.0002 (2)	−0.0016 (3)
C10	0.0389 (6)	0.0525 (8)	0.0134 (3)	−0.0133 (6)	−0.0001 (4)	0.0069 (4)
C11	0.0218 (4)	0.0125 (3)	0.0380 (5)	0.0005 (3)	0.0094 (4)	0.0044 (3)
C12	0.0348 (6)	0.0154 (4)	0.0684 (10)	0.0063 (4)	0.0162 (7)	0.0115 (5)
C13	0.0208 (4)	0.0259 (5)	0.0474 (7)	−0.0059 (3)	0.0048 (4)	−0.0147 (5)
C14	0.0215 (5)	0.0388 (7)	0.0620 (10)	−0.0095 (5)	0.0094 (5)	−0.0147 (6)
O1	0.0155 (2)	0.0186 (3)	0.0138 (2)	−0.00277 (19)	0.00266 (18)	−0.0023 (2)
O2	0.0133 (2)	0.0123 (2)	0.0167 (2)	0.00077 (17)	−0.00018 (18)	−0.00223 (18)
O3	0.0224 (3)	0.0169 (2)	0.0123 (2)	−0.0007 (2)	0.00171 (19)	0.00366 (19)
O4	0.0152 (2)	0.0107 (2)	0.0225 (3)	0.00143 (18)	0.0041 (2)	0.00108 (19)
O6	0.0152 (3)	0.0243 (3)	0.0308 (4)	−0.0012 (2)	−0.0009 (2)	−0.0068 (3)
O7	0.0162 (3)	0.0204 (3)	0.0294 (3)	0.0049 (2)	−0.0024 (2)	−0.0041 (3)
O8	0.0306 (4)	0.0338 (4)	0.0194 (3)	0.0082 (3)	−0.0021 (3)	−0.0102 (3)
O9	0.0289 (4)	0.0214 (3)	0.0756 (8)	0.0018 (3)	0.0261 (5)	0.0143 (5)
O10	0.0344 (5)	0.0249 (5)	0.1202 (15)	−0.0055 (4)	0.0250 (7)	−0.0233 (7)
S1	0.01293 (7)	0.01785 (8)	0.01451 (7)	0.00059 (6)	0.00218 (6)	0.00388 (6)
S2	0.01376 (8)	0.03284 (13)	0.02396 (10)	0.00211 (8)	−0.00070 (7)	0.01238 (9)
C15	0.0137 (3)	0.0196 (3)	0.0136 (3)	−0.0015 (2)	−0.0003 (2)	0.0036 (2)
N1	0.0150 (3)	0.0284 (4)	0.0154 (3)	−0.0016 (3)	0.0019 (2)	0.0076 (3)
C16	0.0145 (3)	0.0331 (5)	0.0235 (4)	0.0011 (3)	0.0047 (3)	0.0061 (3)
C17	0.0257 (5)	0.0479 (7)	0.0270 (5)	−0.0008 (5)	0.0029 (4)	0.0226 (5)

### 2,3,4,6-Tetra-O-acetyl-1-[(dimethylcarbamothioyl)sulfanyl]-β-D-galactopyranose Geometric parameters (Å, º)

**Table:**

C1—O1	1.4216 (11)	N1—C17	1.4631 (14)
C1—C2	1.5378 (11)	N1—C16	1.4655 (13)
C1—S1	1.7959 (8)	C1—H1	1.0000
C2—O2	1.4339 (10)	C2—H2	1.0000
C2—C3	1.5255 (11)	C3—H3	1.0000
C3—O3	1.4358 (10)	C4—H4	1.0000
C3—C4	1.5226 (11)	C5—H5	1.0000
C4—O4	1.4419 (10)	C6—H6A	0.9900
C4—C5	1.5234 (11)	C6—H6B	0.9900
C5—O1	1.4290 (11)	C8—H8A	0.9800
C5—C6	1.5209 (12)	C8—H8B	0.9800
C6—O6	1.4365 (13)	C8—H8C	0.9800
C7—O7	1.2079 (11)	C10—H10A	0.9800
C7—O2	1.3585 (11)	C10—H10B	0.9800
C7—C8	1.4936 (14)	C10—H10C	0.9800
C9—O8	1.2045 (15)	C12—H12A	0.9800
C9—O3	1.3639 (12)	C12—H12B	0.9800
C9—C10	1.4966 (15)	C12—H12C	0.9800
C11—O9	1.2055 (14)	C14—H14A	0.9800
C11—O4	1.3513 (12)	C14—H14B	0.9800
C11—C12	1.4968 (16)	C14—H14C	0.9800
C13—O10	1.2015 (18)	C16—H16A	0.9800
C13—O6	1.3475 (16)	C16—H16B	0.9800
C13—C14	1.4989 (18)	C16—H16C	0.9800
S1—C15	1.7877 (9)	C17—H17A	0.9800
S2—C15	1.6698 (9)	C17—H17B	0.9800
C15—N1	1.3327 (12)	C17—H17C	0.9800
			
O1—C1—C2	109.30 (7)	C2—C3—H3	109.3
O1—C1—S1	108.81 (5)	O4—C4—H4	109.9
C2—C1—S1	110.40 (6)	C3—C4—H4	109.9
O2—C2—C3	106.99 (6)	C5—C4—H4	109.9
O2—C2—C1	109.76 (6)	O1—C5—H5	109.0
C3—C2—C1	107.53 (6)	C6—C5—H5	109.0
O3—C3—C4	107.51 (6)	C4—C5—H5	109.0
O3—C3—C2	109.82 (7)	O6—C6—H6A	110.4
C4—C3—C2	111.46 (6)	C5—C6—H6A	110.4
O4—C4—C3	108.79 (7)	O6—C6—H6B	110.4
O4—C4—C5	108.90 (6)	C5—C6—H6B	110.4
C3—C4—C5	109.42 (6)	H6A—C6—H6B	108.6
O1—C5—C6	105.43 (7)	C7—C8—H8A	109.5
O1—C5—C4	111.03 (7)	C7—C8—H8B	109.5
C6—C5—C4	113.18 (7)	H8A—C8—H8B	109.5
O6—C6—C5	106.69 (8)	C7—C8—H8C	109.5
O7—C7—O2	123.08 (8)	H8A—C8—H8C	109.5
O7—C7—C8	125.92 (9)	H8B—C8—H8C	109.5
O2—C7—C8	110.99 (8)	C9—C10—H10A	109.5
O8—C9—O3	123.52 (9)	C9—C10—H10B	109.5
O8—C9—C10	126.22 (11)	H10A—C10—H10B	109.5
O3—C9—C10	110.25 (11)	C9—C10—H10C	109.5
O9—C11—O4	123.65 (10)	H10A—C10—H10C	109.5
O9—C11—C12	125.51 (10)	H10B—C10—H10C	109.5
O4—C11—C12	110.83 (9)	C11—C12—H12A	109.5
O10—C13—O6	123.22 (12)	C11—C12—H12B	109.5
O10—C13—C14	126.00 (14)	H12A—C12—H12B	109.5
O6—C13—C14	110.77 (11)	C11—C12—H12C	109.5
C1—O1—C5	111.25 (6)	H12A—C12—H12C	109.5
C7—O2—C2	117.64 (7)	H12B—C12—H12C	109.5
C9—O3—C3	117.41 (7)	C13—C14—H14A	109.5
C11—O4—C4	117.07 (7)	C13—C14—H14B	109.5
C13—O6—C6	118.06 (9)	H14A—C14—H14B	109.5
C15—S1—C1	101.77 (4)	C13—C14—H14C	109.5
N1—C15—S2	124.18 (7)	H14A—C14—H14C	109.5
N1—C15—S1	112.09 (7)	H14B—C14—H14C	109.5
S2—C15—S1	123.71 (5)	N1—C16—H16A	109.5
C15—N1—C17	119.92 (9)	N1—C16—H16B	109.5
C15—N1—C16	123.38 (8)	H16A—C16—H16B	109.5
C17—N1—C16	116.62 (8)	N1—C16—H16C	109.5
O1—C1—H1	109.4	H16A—C16—H16C	109.5
C2—C1—H1	109.4	H16B—C16—H16C	109.5
S1—C1—H1	109.4	N1—C17—H17A	109.5
O2—C2—H2	110.8	N1—C17—H17B	109.5
C3—C2—H2	110.8	H17A—C17—H17B	109.5
C1—C2—H2	110.8	N1—C17—H17C	109.5
O3—C3—H3	109.3	H17A—C17—H17C	109.5
C4—C3—H3	109.3	H17B—C17—H17C	109.5
			
O1—C1—C2—O2	176.61 (6)	C8—C7—O2—C2	−178.12 (9)
S1—C1—C2—O2	−63.74 (7)	C3—C2—O2—C7	−142.58 (7)
O1—C1—C2—C3	60.57 (8)	C1—C2—O2—C7	101.04 (8)
S1—C1—C2—C3	−179.78 (5)	O8—C9—O3—C3	7.42 (14)
O2—C2—C3—O3	68.17 (8)	C10—C9—O3—C3	−172.48 (9)
C1—C2—C3—O3	−173.98 (6)	C4—C3—O3—C9	127.59 (8)
O2—C2—C3—C4	−172.80 (6)	C2—C3—O3—C9	−110.98 (8)
C1—C2—C3—C4	−54.95 (8)	O9—C11—O4—C4	−1.10 (18)
O3—C3—C4—O4	53.85 (8)	C12—C11—O4—C4	179.96 (11)
C2—C3—C4—O4	−66.55 (8)	C3—C4—O4—C11	−113.01 (9)
O3—C3—C4—C5	172.72 (7)	C5—C4—O4—C11	127.80 (9)
C2—C3—C4—C5	52.32 (8)	O10—C13—O6—C6	5.4 (2)
O4—C4—C5—O1	64.22 (9)	C14—C13—O6—C6	−173.27 (11)
C3—C4—C5—O1	−54.58 (9)	C5—C6—O6—C13	127.99 (11)
O4—C4—C5—C6	−54.12 (9)	O1—C1—S1—C15	−84.52 (6)
C3—C4—C5—C6	−172.92 (7)	C2—C1—S1—C15	155.53 (6)
O1—C5—C6—O6	179.61 (8)	C1—S1—C15—N1	170.41 (7)
C4—C5—C6—O6	−58.86 (10)	C1—S1—C15—S2	−11.04 (7)
C2—C1—O1—C5	−65.67 (8)	S2—C15—N1—C17	−3.08 (15)
S1—C1—O1—C5	173.70 (6)	S1—C15—N1—C17	175.46 (10)
C6—C5—O1—C1	−174.41 (7)	S2—C15—N1—C16	−179.75 (9)
C4—C5—O1—C1	62.66 (9)	S1—C15—N1—C16	−1.21 (13)
O7—C7—O2—C2	1.12 (13)		

### 2,3,4,6-Tetra-O-acetyl-1-[(dimethylcarbamothioyl)sulfanyl]-β-D-galactopyranose Hydrogen-bond geometry (Å, º)

**Table:**

*D*—H···*A*	*D*—H	H···*A*	*D*···*A*	*D*—H···*A*
C4—H4···O7^i^	1.00	2.45	3.2096 (11)	132
C8—H8*B*···O8^ii^	0.98	2.41	3.3157 (15)	154
C16—H16*C*···O10^iii^	0.98	2.44	3.1795 (18)	132
C17—H17*B*···O1^iv^	0.98	2.55	3.3558 (13)	139
C1—H1···S2	1.00	2.56	3.1175 (8)	115
C8—H8*A*···O8^v^	0.98	2.66	3.3384 (15)	127

Symmetry codes: (i) *x*+1, *y*, *z*; (ii) *x*−1, *y*, *z*; (iii) *x*−1, *y*−1, *z*; (iv) −*x*, *y*−1/2, −*z*+1/2; (v) *x*−1/2, −*y*+1/2, −*z*.
